# A National Survey on the Implementation Status and Current Quality Monitoring Practices of Nutrition Nursing Clinics in China

**DOI:** 10.1002/nop2.70671

**Published:** 2026-07-05

**Authors:** Xing Zeng, Xuan Ni, Hui Liu, Zhili Shen, Yajing Gu, Ai Li, Xiuya Ren, Changdi Li

**Affiliations:** ^1^ School of Nursing Nanjing Medical University Nanjing China; ^2^ Department of Gastrointestinal and Pancreatic Surgery The Affiliated Jiangning Hospital of Nanjing Medical University Nanjing China; ^3^ Chinese Hospital Reform and Development Institute Nanjing University Nanjing China; ^4^ Department of Orthopedics The Affiliated Jiangning Hospital of Nanjing Medical University Nanjing China; ^5^ Department of Urology The Affiliated Jiangning Hospital of Nanjing Medical University Nanjing China; ^6^ Department of Nursing The Affiliated Jiangning Hospital of Nanjing Medical University Nanjing China; ^7^ Department of Nursing The First Affiliated Hospital of Guizhou University of Traditional Chinese Medicine Guiyang China

**Keywords:** cross‐sectional survey, Donabedian framework, implementation status, nutrition nursing clinic, quality monitoring landscape

## Abstract

**Aim:**

This national, cross‐sectional survey aimed to describe the current implementation status of Nutrition Nursing Clinics (NNCs) in China and to map the landscape of quality monitoring indicators in use, thereby establishing a foundational evidence base for future service optimisation and the development of standardised evaluation tools.

**Design:**

A nationwide cross‐sectional survey.

**Methods:**

Conducted from August to October 2024, the study involved 164 randomly sampled public hospitals. Data were collected via a validated 32‐item questionnaire, developed through a systematic process of literature review, expert consultation (*n* = 10) and pilot testing. The questionnaire was structured into four domains: (I) General Hospital Demographics, (II) Current Operational Status of the Nutrition Clinic, (III) Current Use of Quality Monitoring Indicators (categorised by the Donabedian Structure‐Process‐Outcome Framework) and (IV) Recommendations for NNC Development. Descriptive statistics were employed for data analysis.

**Results:**

The valid response rate was 68.9% (113/164). Key findings across the four survey domains were: (1) Establishment and Basic Profile: Among the respondents, 37 hospitals (32.7%) had established NNCs, with 75.7% initiated within the past 5 years. (2) Operational Characteristics: NNCs were predominantly in tertiary hospitals (70.3%), operated 1–3 days per week (78.4%) and were mostly managed by nursing departments (70.3%). Daily patient volume was typically ≤ 15 (75.6%). (3) Quality Monitoring Landscape: A pooled inventory of 66 distinct monitoring indicators was identified, revealing significant heterogeneity in practice. Monitoring focused on structural elements (e.g., personnel qualifications) and initial processes (e.g., assessment), with less emphasis on care continuity, interdisciplinary collaboration and patient‐reported outcomes. (4) Challenges and Suggestions: The reported challenges primarily focused on systemic and operational barriers. The most prominent issues included the absence of billing items for nutrition techniques (67.6%) and insufficient insurance coverage (59.5%). Additional challenges included limited prescription authority for nurses and practical difficulties in follow‐up management. Furthermore, participating hospitals proposed specific suggestions for development, such as enhancing feasibility through strengthened publicity and interdepartmental collaboration, as well as ensuring financial sustainability to support operations by establishing upstream and downstream cooperative relationships with other clinical departments.

**Conclusion:**

This study provides the first national profile of NNC implementation in China. The findings reveal a field characterised by a low establishment rate, concentrated service delivery and a complete absence of standardised quality monitoring. A core contribution of this work is the systematic survey and compilation of a descriptive inventory of 66 quality monitoring indicators currently in use, which we categorised and analysed according to the Donabedian framework. This inventory reflects the highly heterogeneous and uncoordinated nature of current evaluation practices. This study provides the necessary foundation to inform the future development of a standardised, contextually relevant quality evaluation framework for NNCs.

**No Patient or Public Contribution:**

This study analysed anonymised institutional data. No direct patient or public involvement occurred.

## 1 Introduction

The National Health Commission of China's 2022 issuance of the ‘Guidelines for the Construction and Management of Clinical Nutrition Departments’ (National Health Commission of the People's Republic of China 2022a) marked a pivotal advancement in standardising professional practice by authorising qualified nutrition specialists to implement comprehensive nutritional services, including consultation, screening/assessment, enteral nutrition formulation, medical dietary preparation and patient education. As critical platforms for executing nutritional support interventions, NNCs serve dual functions in therapeutic care delivery and health promotion, playing an indispensable role in optimising patient nutritional status, preserving physiological functions and elevating quality of life metrics. This emerging field has attracted considerable attention in healthcare quality evaluation amid rising public health awareness. However, the delivery model of NNCs in China remains immature and faces persistent systemic challenges. Huang et al. (2016) proposed an organisational architecture featuring nutrition support nursing working groups under the collaborative leadership of nursing administration and outpatient departments, structurally integrated with hospital‐based clinical nutrition centres. Nevertheless, practical implementation reveals critical gaps, including workforce shortages in certified practitioners, variability in service standardisation and inadequate health literacy interventions, compounded by the absence of unified national protocols for service specifications and quality control mechanisms.

Since its introduction in 1966, Avedis Donabedian's three‐dimensional quality structure model, which evaluates healthcare quality through the dimensions of structure, process and outcome, has served as the foundational framework guiding national discourse on this subject (Donabedian 1966). Within the field of nursing, quality evaluation constitutes a pivotal component of nursing quality management. Evaluation indicators provide the essential basis for nursing administration, establish the standards for assessing nursing services and guide clinical nursing practice. Consequently, establishing a systematic, scientific and advanced nursing quality evaluation system is crucial for enhancing both the quality of care and the standard of nursing management. However, the development of such an evaluation system necessitates a deep understanding of the local clinical context. In China, a nationwide overview of NNCs—encompassing their implementation status, operational models and current quality monitoring practices—remains absent. This study aims to address this gap by systematically investigating the landscape of NNC implementation and cataloguing the specific quality monitoring indicators currently employed across the structure, process and outcome dimensions. The resulting profile establishes the necessary empirical foundation for the future development of a contextualised quality evaluation framework based on the Donabedian model.

## 2 Research Aim and Objectives

The study aims to address the following research question: ‘What is the current implementation status and quality monitoring landscape of NNCs in China?’ To achieve this, it targets two primary objectives, namely:

- To profile the nationwide implementation status of NNCs, including their establishment rates, operational characteristics and the primary challenges faced in their delivery.
- To identify the quality monitoring indicators currently employed by established NNCs across the dimensions of the Donabedian framework.

## 3 Methodology

### 3.1 Study Design and Overall Approach

This study employed a national cross‐sectional survey design. From August 1 to October 31, 2024, a total of 164 public medical institutions were randomly selected to investigate the operational status of NNCs and to map the landscape of their quality monitoring indicators. The methodology comprised three sequential phases: (1) the systematic development and validation of a survey instrument; (2) nationwide data collection; and (3) statistical analysis.

### 3.2 Instrument Development and Validation

The initial item pool and questionnaire domains were derived from a comprehensive literature review on nurse‐led clinics, nutrition support and healthcare quality indicators. This review shaped the preliminary survey structure, ensuring that the quality monitoring indicators aligned with the Donabedian framework.

#### 3.2.1 Literature Review and Item Generation

##### 3.2.1.1 Database Selection

The following databases were systematically searched: China National Knowledge Infrastructure (CNKI), Wanfang Medical Network, VIP Full‐Text Database, Cochrane Library, PubMed, Embase, Medline and Web of Science. This study systematically retrieved evidence from January 2014 to April 2024.

##### 3.2.1.2 Search Terms

A combination of Medical Subject Headings (MeSH) and free‐text terms was employed to optimise recall and precision. MeSH terms included: ‘Office Nursing’, ‘Specialties, Nursing’, ‘Quality Indicators, Health Care’, ‘Quality Assurance, Health Care’, ‘Health Care Surveys’. Free‐text keywords included: Titles/abstracts were searched using phrases like ‘Outpatient Nursing’, ‘Nursing Clinics’, ‘Nursing Clinic’, ‘Clinical Nutrition Nursing’, ‘Evaluation index’, ‘Quality index’, ‘Quality Evaluation’, ‘Nursing quality’, ‘Quality Control’, ‘Current Status’, ‘Current State’, ‘Development Status’, ‘Implementation Status’, ‘Present Situation’, ‘Operational Status’.

PubMed Search Strategy: ((‘Office Nursing’[Mesh] OR ‘Specialties, Nursing’[Mesh]) OR (‘Outpatient Nursing’[tiab] OR ‘Nursing Clinics’[tiab] OR ‘Nursing Clinic’[tiab] OR ‘Clinical Nutrition Nursing’[tiab])) AND ((‘Quality Indicators, Health Care’[Mesh] OR ‘Quality Assurance, Health Care’[Mesh]) OR (‘Evaluation index’[tiab] OR ‘Quality index’[tiab] OR ‘Quality Evaluation’[tiab] OR ‘Nursing Quality’[tiab] OR ‘Quality Control’[tiab])) AND ((‘Health Care Surveys’[Mesh]) OR (‘Current Status’[tiab] OR ‘Current State’[tiab] OR ‘Development Status’[tiab] OR ‘Implementation Status’[tiab] OR ‘Present Situation’[tiab] OR ‘Operational Status’[tiab])).

##### 3.2.1.3 Inclusion and Exclusion

Inclusion Criteria: ① Publication Type: Original studies, evidence syntheses, guidelines, consensus statements and methodological studies on quality indicator development/validation. ② Subject Focus: Studies on nurse‐led outpatient clinics, specifically addressing quality assessment, implementation status, or metrics; priority given to those focused on clinical nutrition nursing or nutrition support. ③ Conceptual Framework: Studies utilising or pertinent to established quality frameworks, especially the Donabedian structure‐process‐outcome model. ④ Population/Context: Outpatient settings with nursing as a primary component, involving stakeholders such as nurses or patients.

Exclusion Criteria: ① Publication Type: Non‐peer‐reviewed literature. ② Topic Irrelevance: Studies limited to inpatient settings, general clinic quality without a nursing focus, or those not addressing quality evaluation or implementation. ③ Inaccessibility: Articles whose full text could not be retrieved.

##### 3.2.1.4 Literature Screening

The literature search was performed independently by two investigators. After duplicate removal with CNKI Research Assistant, records were screened based on titles, abstracts and keywords. Additional studies were identified by snowballing the references of included articles. Conflicts during screening were resolved through discussion or arbitration by a third investigator.

##### 3.2.1.5 Literature Synthesis and Development of the Survey Framework for the Current Status of NNCs

**FIGURE 1:**
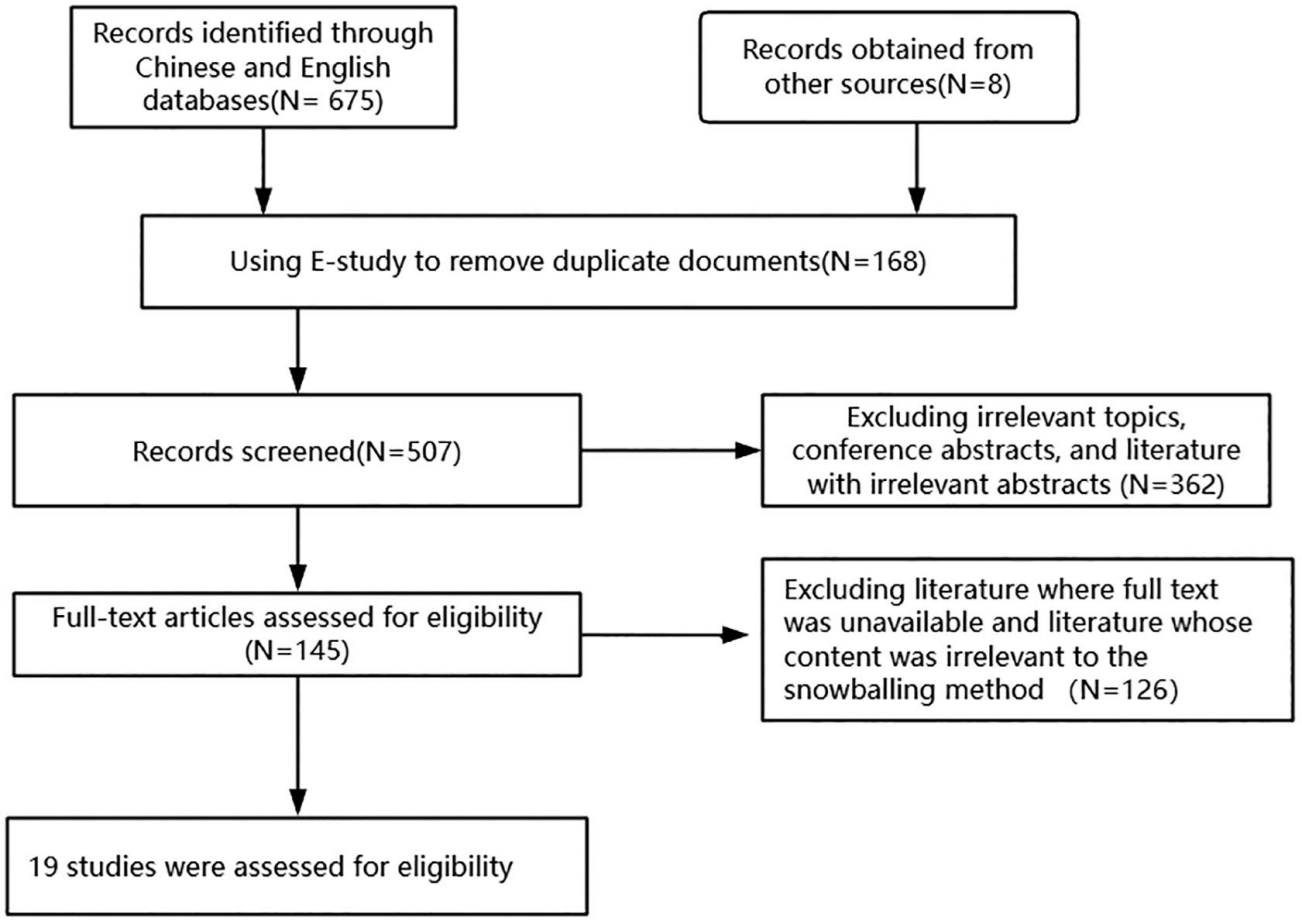
Flowchart of literature screening.

The included literature was discussed and analyzed and the findings were summarised to develop a survey framework for the current status of NNCs. A total of 683 documents were retrieved through the search and after removing duplicates and screening, 19 documents (Shi et al. 2019; Tian et al. 2019, 2025, 2018; Lin et al. 2019; Kiss et al. 2020; Liu et al. 2021; Pleh et al. 2021; Swan et al. 2017; Benítez Brito et al. 2021; Sun et al. 2023; Wang et al. 2021, 2020, 2022; Muscaritoli et al. 2021; Wang, Cheng, and Ye 2019; Gan et al. 2021; Wang, Liu, et al. 2019; Zatko et al. 2021) were finally included, using the snowball method. The process of literature screening is detailed in Figure 1.

##### 3.2.1.6 Literature Summary

Based on the included literature, a discussion and analysis were conducted and the survey content was derived through categorisation and summarisation, as detailed in Table 1.

**TABLE 1:** Survey framework: NNC Operations and quality indicator utilisation.

Major category/dimension	Specific survey points/indicators	Literature sources
Basic Implementation Status	Years of Operation	Shi et al. (2019)
	Number of Patient Visits	Shi et al. (2019)
	Nature of Position and Number of Nurses on Duty	Tian et al. (2019)
	Operational Model (Doctor‐Nurse Collaboration/Independent Nurse‐led Clinic)	Lin et al. (2019), Shi et al. (2019), Kiss et al. (2020)
	Mode of Consultation (In‐person/Internet+)	Lin et al. (2019), Liu et al. (2021)
	Dietitian/Physician Configuration in the Nutrition Clinic	Tian et al. (2025), Pleh et al. (2021), Swan et al. (2017), Benítez Brito et al. (2021), Sun et al. (2023)
	Registration Method	Lin et al. (2019), Liu et al. (2021)
	Cumulative Weekly Opening Hours	Liu et al. (2021)
Environment and Facilities	Clinic Layout and Environment	Wang et al. (2021), Liu et al. (2021), Tian et al. (2018), Wang et al. (2020)
	Completeness of Basic Clinic Equipment, e.g., specialised nutritional assessment and body composition devices (e.g., BIA, handgrip dynamometer)	Liu et al. (2021), Tian et al. (2018), Swan et al. (2017), Muscaritoli et al. (2021), Wang et al. (2022)
	Patient Privacy Protection	Muscaritoli et al. (2021)
Personnel Qualifications and Training	Professional Title	Shi et al. (2019), Wang, Cheng, and Ye (2019)
	Years of Work Experience	Lin et al. (2019), Gan et al. (2021)
	Years of Specialised Nutrition Work and Certification	Kiss et al. (2020), Benítez Brito et al. (2021)
	Educational Background	Lin et al. (2019), Tian et al. (2018)
	Assessment, Training and Advanced Studies (particularly in clinical nutrition)	Lin et al. (2019), Wang, Liu, et al. (2019), Zatko et al. (2021), Sun et al. (2023)
Nutrition Care Techniques and Management	Application of Nutritional Risk Screening and Assessment Tools (e.g., NRS‐2002, PG‐SGA)	Swan et al. (2017), Benítez Brito et al. (2021), Muscaritoli et al. (2021)
	Implementation of Medical Nutrition Therapy (MNT)	Swan et al. (2017), Benítez Brito et al. (2021)
	Development of Individualised Nutrition Care Plans and Follow‐up Management	Kiss et al. (2020), Muscaritoli et al. (2021), Wang et al. (2022)
Rules and Regulations	Establishment of Systems (Infection Control, Adverse Event Reporting, Job Responsibilities, Appointment Management, etc.)	Wang et al. (2021), Gan et al. (2021), Sun et al. (2023), Wang et al. (2022)
Patient Outcomes	Improvement in Nutritional Status	Wang et al. (2022)
	Quality of Life Improvement	Sun et al. (2023)
Patient and Healthcare Provider Satisfaction	Patient Satisfaction	Wang et al. (2020)
	Doctor/Nurse Job Satisfaction	Wang et al. (2020)
Difficulties and Challenges	Clinic Promotion and Patient Awareness Rate	Tian et al. (2025), Liu et al. (2021)
	Problems Encountered During Implementation	Lin et al. (2019), Shi et al. (2019), Kiss et al. (2020)

#### 3.2.2 Expert Meetin*g* and Content Validity

##### 3.2.2.1 Expert Panel Review: Methodology, Composition and Procedure

An expert meeting was held to review and refine the initial questionnaire draft. Experts in clinical nutrition nursing (management, specialised practice) and clinical nutrition were invited, with inclusion criteria requiring a bachelor's degree or higher, associate senior title or above and over 10 years of relevant experience. Preparatory materials were distributed via WeChat prior to the meeting. During the session, researchers presented the draft via PowerPoint and facilitated a structured discussion on the content's appropriateness, necessity and feasibility, with proceedings formally recorded. Feedback was subsequently collated and used to implement substantive revisions to the survey instrument.

##### 3.2.2.2 Expert Positive Coefficient, Degree of Authority and Degree of Coordination of Opinions

Ten experts participated in the questionnaire design. All attended the meeting and provided revision suggestions, yielding a 100% response rate. The panel was predominantly female (70%), aged 36–54 years, with 10–31 years of professional experience. It consisted of four nursing managers, three clinical nutrition specialist nurses and three clinical nutrition physicians. Regarding highest earned degrees, five held a master's, four a bachelor's and one a doctorate; all possessed an associate senior professional title or higher. The expert authority coefficient (Cr) was calculated using the formula Cr = (Ca + Cs)/2, based on the self‐assessed results of the judgement basis (Ca) and the expert familiarity (Cs). In this study, Ca = 0.90, Cs = 0.82, resulting in a Cr of 0.86.

##### 3.2.2.3 Summary of Expert Opinions

- Modification Suggestions for General Information: ① Change ‘Province (Autonomous Region, Municipality) where the hospital is located’ to a fill‐in‐the‐blank ‘Your Hospital Name’ to reduce ambiguity and facilitate data linkage. ② Update the bed capacity statistical time point from ‘as of August 31, 2024’ to ‘as of now’ for real‐time data accuracy. ③ Optimise question order (e.g., place ‘Hospital Type’ first) and simplify options (e.g., remove ‘Ungraded’) to enhance fluency and reduce redundancy.
- Modification Suggestions for the Implementation Status of NNCs: ① Add ‘Does your hospital operate nutrition‐related outpatient services involving nurse participation?’ as the first question in Part II. ② Add ‘Internet + Home Visit’ as a consultation method option. ③ Add ‘Ward availability situation of the nutrition clinic’ (e.g., independent/borrowed wards). ④ Add ‘Average daily patient volume’ as a quantitative efficiency indicator. ⑤ Add ‘Chronic disease management’ and ‘Weight management’ to the services list. ⑥ Add ‘Bioelectrical Impedance Analyzer (BIA)’ and ‘Dietary record tools’ to the equipment list. ⑦ Add ‘Games and interactive activities’ to health education methods. ⑧ Add ‘Situation of nurses' prescription rights’ (e.g., independent non‐drug orders). ⑨ To the problems list, add: few patients, poor multidisciplinary collaboration, difficulty in personalised services, challenges in follow‐up, insufficient IT support and cultural/dietary habit differences.
- Modification Suggestions for Personnel Situation of NNCs: ① Change personnel inquiry from current ‘distribution’ to ‘access requirements’ (e.g., title, experience) to investigate hiring thresholds. ② Delete inquiry on nurses' general work years; only investigate ‘work years in nutrition nursing’. Segment experience options precisely (e.g., ‘1–3 years’). ③ Delete the requirement for external training duration, as holding a recognised qualification certificate is deemed sufficient.
- Modification Suggestions for Nursing Quality Monitoring Indicators Status: ① Structural Dimension: Add ‘Functionality/availability of consultation room equipment’, ‘Patient safety/privacy protection measures’ and ‘Effectiveness of patient record management systems’. ② Process Dimension: Add indicators for: Interdisciplinary collaboration; Patient training in knowledge/behaviour/self‐management; Frequency/quality of follow‐up; Regularity/validity of monitoring protocols. Replace ‘assessment rate’ with ‘Comprehensiveness of nutritional assessment’. ③ Outcome Dimension: Add indicators: ‘Complication rate’, ‘Quality of life improvement rate’, ‘Monitoring of quality control sensitive indicators’, ‘Economic benefits’, ‘Diagnostic service coverage’. Change ‘Therapeutic effect on patient diseases’ to ‘Nutritional status improvement rate’.

##### 3.2.2.4 Content Validity Evaluation

Following the expert review, the content validity of the questionnaire was quantitatively assessed. Experts rated item relevance and appropriateness and ratings were used to calculate the Content Validity Index (CVI). The Scale‐level CVI (S‐CVI) was 0.92 and all Item‐level CVI (I‐CVI) values exceeded 0.78, confirming excellent content validity.

#### 3.2.3 Pilot Testing and Reliability Assessment

To evaluate the psychometric properties of the questionnaire, a pilot test was conducted in three tertiary hospitals with established nutrition clinics (*n* = 3), targeting the heads of nursing departments or outpatient departments. For test–retest reliability assessed over a two‐week interval, the intraclass correlation coefficient (ICC) was 0.91 (95% CI: 0.85–0.95), which is above the benchmark of 0.75 for excellent reliability. These findings indicate that the questionnaire possesses good reliability.

Based on feedback from the pilot testing, two primary areas for refinement were identified. First, regarding item wording, ambiguity was noted in certain professional terms. For instance, in Item 21, respondents found it difficult to accurately distinguish between the options ‘Technical Operating Protocols for Nutrition Care’ and ‘Nutrition Assessment and Management Protocols’. To address this, we added concise illustrative examples to such potentially confusing items. Specifically, ‘Technical Operating Protocols for Nutrition Care’ was elaborated as ‘for example, Enteral Nutrition Tube Feeding Protocol, Standards for Nutrition Pump Use, etc.’ to facilitate more precise selection. Second, a suggestion was made to optimise the survey's skip logic. Originally, respondents selecting ‘No’ for Item 6 were directed to the end of the questionnaire. It was proposed that for hospitals not currently operating such clinics, brief information on their ‘planned implementation timeline’ or ‘primary perceived barriers’ could be collected. However, as the core focus of this study is to profile institutions with existing nutrition clinics, the fundamental skip logic was retained.

#### 3.2.4 Final Questionnaire

Following expert review and research team consensus, the final questionnaire was established. It comprises 32 validated items across four domains: (I) General Hospital Information, (II) Current Operational Status of the Nutrition Clinic, (III) Current Use of Quality Monitoring Indicators Categorised by the Donabedian Structure‐Process‐Outcome Framework and (IV) Recommendations for the Development of the Nutrition Nursing Clinic. The questionnaire is designed to be completed by authorised personnel, specifically nursing administrators or outpatient department leads.

### 3.3 Survey Methods and Quality Control

A standardised survey protocol was implemented using electronic questionnaires distributed via the Wenjuanxing platform. A coordinator monitored responses in real time and conducted telephone follow‐ups for incomplete or inconsistent data. Invitations were sent to 164 institutions, with 113 providing valid responses (68.9% response rate). Data integrity was ensured through dual independent verification, where two researchers checked the data files exported from the Wenjuanxing platform.

### 3.4 Statistical Methods

Data underwent dual‐entry verification in Microsoft Excel 2019 with logic‐error screening, followed by statistical analyses performed using SPSS 22.0. Categorical variables were analysed using frequency distributions, constituent ratios and rates to characterise institutional and operational patterns. Importantly, the Donabedian (Structure‐Process‐Outcome) framework was employed post hoc as an analytical and organising schema. After data collection, this framework was used to categorise and thematically present the exhaustive list of quality monitoring indicators that were self‐reported by the 37 NNCs as currently in use within their clinics.

## 4 Results

### 4.1 General Information of Surveyed Hospitals

A total of 164 questionnaires were distributed and collected, yielding 113 valid responses, representing a valid response rate of 68.9%. The general information of the 113 surveyed hospitals is summarised in Table 2. The surveyed hospitals were predominantly located in the eastern region (64 hospitals, 56.6%), of which 20 (31.3% of eastern hospitals) operated Nurse‐Led Clinics (NNCs). Meanwhile, 49 hospitals (43.4%) were distributed across the western, central and northeastern regions. The survey covered 41 cities, including: Nanjing, Guiyang, Wuxi, Suzhou, Wuhan, Jinan, Ningnan, Qingdao, Changzhou, Chengdu, Kunming, Hefei, Pingtang, Ningbo, Chuzhou, Wuhu, Shanghai, Jining, Qingyuan, Neijiang, Tongling, Shenyang, Handan, Qujing, Ziyang, Lezhi, Zigong, Chongqing, Zhengzhou, Duyun, Zhoukou, Xi'an, Laifeng, Haikou, Jiaozuo, Taizhou, Zunyi and Hexian. Among the 113 medical institutions surveyed, 37 (32.7%) had established specialised NNCs with nurse‐led consultation services.

**TABLE 2:** General information of the surveyed hospitals (*n* = 113).

Project name	Frequency	Proportion (%)
Hospital Grade		
Tier III Grade A Hospital	86	76.1
Tier III Grade B Hospital	15	13.3
Below Tier II Hospital	12	10.6
Hospital Type		
Western Medicine Hospital	57	50.4
Integrated Traditional Chinese and Western Medicine Hospital	22	19.5
Traditional Chinese Medicine Hospital	12	10.6
Specialised Hospital	19	16.8
Community Hospital	3	2.7
Teaching Hospital Status		
Non‐teaching Hospital	13	11.5
Teaching Hospital	100	88.5

### 4.2 General Information of Medical Institutions With NNCs

Nutritional nursing outpatient services in China are predominantly concentrated in large academic medical centres, with 70.3% of providers being Tier III Grade A hospitals. Western medicine institutions deliver most services (62.2%), far exceeding traditional Chinese medicine (2.7%) or integrated models (16.2%). Over 89% operate as teaching hospitals and 78.3% have ≥ 1000 beds, highlighting a service concentration in high‐capacity, research‐oriented facilities. This distribution suggests limited accessibility for patients in smaller hospitals (21.6% with ≤ 500 beds) or non‐academic settings (Table 3).

**TABLE 3:** General information of medical institutions offering nutritional nursing outpatient services (*n* = 37).

Project name	Frequency	Proportion (%)
Hospital Grade		
Tier III Grade A Hospital	26	70.3
Tier III Grade B Hospital	7	18.9
Below Tier II Hospital	4	10.8
Hospital Type		
Western Medicine Hospital	23	62.2
Integrated Traditional Chinese and Western Medicine Hospital	6	16.2
Traditional Chinese Medicine Hospital	1	2.7
Specialised Hospital	7	18.9
Whether it is a Teaching Hospital		
No	4	10.8
Yes	33	89.2
Number of Hospital Beds		
≤ 200 beds	3	8.1
200–499 beds	2	5.4
500–999 beds	3	8.1
1000–1499 beds	8	21.6
1500–1999 beds	5	13.5
≥ 2000 beds	16	43.2

### 4.3 Status of NNCs

The duration of NNCs ranged from less than 1 year to 12 years. NNCs established within the last 5 years accounted for 75.7% of the total. Full‐time NNC nurses accounted for 51.4%. In terms of management models, the Nursing Department was mostly in charge (70.3%). Only 10.8% provided home services. The consultation format was mostly a combination of medical and nursing staff (78.4%), with collaborative medical orders accounting for 54.0%. NNCs had a wide range of nutrition assessment tools and content, but faced many challenges, as detailed in Table 4.

**TABLE 4:** Operational characteristics of established NNCs (*n* = 37).

Project name	Frequency	Proportion (%)
Years of Operation		
≤ 5 years	28	75.7
6–9 years	3	8.1
≥ 10 years	6	16.2
Post Forms of Nurses on Duty		
Part‐time	11	29.7
Full‐time	19	51.4
Full‐time & Part‐time	7	18.9
Management Modes		
Coordinated by the Nursing Department	14	37.8
Led by the Department	11	29.7
Coordinated by the Nursing Department & Led by the Department	12	32.4
Outpatient Service Modes		
Outpatient Clinic	18	48.6
Outpatient Clinic & Internet‐based Online Consultation	15	40.5
Outpatient Clinic & Internet‐based Online Consultation & Internet‐based Home Visit	4	10.8
Forms of Outpatient Service		
Nurses on Duty Independently	8	21.6
Joint Outpatient Service by Doctors and Nurses	20	54.1
Joint Outpatient Service by Doctors and Nurses & Nurses on Duty Independently	9	24.3
Situation of Nutritional Outpatient Wards		
Independent Nutritional Department Wards	11	29.7
Occupying Wards of Related Diseases	11	29.7
No Beds in the Nutritional Department	15	40.5
Total Weekly Outpatient Service Hours		
1–3	29	78.4
4–5	6	16.2
6–7	2	5.4
Average Daily Patient Reception Volume		
< 5 Patients	13	35.1
5–15 Patients	15	40.5
16–30 Patients	7	18.9
30–50 Patients	1	2.7
> 50 Patients	1	2.7
Nurses' Prescription Rights		
Cooperative Medical Orders between Doctors and Nurses	17	45.9
Independent Non‐drug Prescription Rights	11	29.7
Independent Drug Prescription Rights	2	5.4
Independent Non‐drug Prescription Rights & Cooperative Medical Orders between Doctors and Nurses	2	5.4
Independent Non‐drug Prescription Rights & Independent Drug Prescription Rights & Cooperative Medical Orders between Doctors and Nurses	1	2.7
None	4	10.8
Sources of Patients		
Patients Directly Registering	23	62.2
Referred by Doctors in Outpatient Clinics	21	56.8
Recommended by Others	15	40.5
Community Promotion	13	35.1
Available Instrument Equipment		
Basal Metabolic Rate Metre	30	81.1
Bioelectrical Impedance Analyser	16	43.2
Weight Scale	34	91.9
Height Measuring Instrument	33	89.2
Skinfold Calliper	21	56.8
Nutrition Consultation Software	16	43.2
Educational Tools	15	40.5
Dietary Record Tools	20	54.1
Used Education and Communication Methods		
Oral Education	35	94.6
Brochures	34	91.9
Video Education	26	70.3
Lectures and Seminars	23	62.2
Telephone or Online Consultation	19	51.4
Social Media and Applications	16	43.2
Games and Interactive Activities	8	21.6
Used Nutritional Assessment Tools		
NRS‐2002	22	59.5
MNA‐SF	15	40.5
PG‐SGA	14	37.8
Anthropometric Measurements (BMI, Circumference Measurement, Skinfold Thickness Measurement)	25	67.6
Limb Function Assessment (Gait Speed, Grip Strength, etc.)	14	37.8
Body Composition Measurement	20	54.1
Basal Metabolic Measurement	15	40.5
Biochemical Indicators	15	40.5
FFQ	4	10.8
MUST	18	48.7
Diet Recall Method	9	24.3
PNMS	7	18.9
SNAQ	6	16.2
Prenatal Nutrition Assessment Questionnaire	6	16.2
Harris‐Benedict Equation	2	5.4
Conducted Services		
Nutritional Screening and Assessment, Nutritional Diagnosis, Implementation and Supervision of Nutritional Therapy	37	100
Providing Medical Diets, EN and PN Recommendations, or Prescriptions as Needed	27	73.0
Standardising Medical Diet Services	22	59.5
Standardising the Use of Foods for Special Medical Purposes	17	46.0
Formulating and Organising the Implementation of Clinical Nutrition‐related Work Specifications in the Institution	19	51.4
Participating in the Consultation of Special, Difficult, Critical and Major Surgery Patients or Joining the MDT Team	14	37.8
Chronic Disease Management: Providing Nutritional Intervention and Management for Patients with Chronic Diseases, such as Diabetes, Hypertension and Hyperlipidemia	18	48.7
Weight Management: Providing Weight Loss or Gain Guidance and Developing Feasible Diet and Exercise Plans	20	54.1
Follow‐up and Evaluation: Regular Follow‐up, Evaluating Nutritional Status and Health Improvement and Adjusting	18	48.7
Undertaking In‐hospital (Out‐of‐hospital) Consultation on Difficult Nutritional Support Complications and Stoma Tube Feeding Nursing	14	37.8
Out‐of‐hospital Nutritional Management: Including Home Nutritional Management and ‘Internet+’ Home Nutritional Practice	11	29.7
Nutritional Nursing Techniques: Such as Establishing and Maintaining EN Pathways, Establishing and Maintaining PN Pathways, etc.	26	70.3
Implementation and Monitoring of Nutritional Support Therapy: Implementing EN and PN, Monitoring Complications and Taking Corresponding Protective Measures	22	59.5
Nutritional Consultation and Education	37	100
Nutritional Management for Special Diseases: For Different Disease States, such as Critical Illness, Tumour, Peri‐operative Period, Liver Disease, etc.	19	51.4
Clinical Nutritional Nursing Management: Such as Evaluating the Core Competence of Nutritional Specialised Nurses and Clinical Practice Teaching, etc.	12	32.4
Existing Problems		
Some Nutritional Nursing Techniques Have No Charging Items	25	67.6
No Independent Non‐drug Prescription Rights	16	43.2
Low Awareness Rate of Nutritional Nursing Outpatient Services	17	46.0
Insufficient Information Technology Support	10	27.0
Charging Items Not Covered by Medical Insurance	22	59.5
Difficulty in Providing Personalised Services	7	18.9
Cultural and Dietary Habit Differences	9	24.3
Few Patients	14	37.8
Poor Multidisciplinary Collaboration	10	27.0
Difficulty in Follow‐up and Continuous Management	15	40.5

Abbreviations: FFQ, Food Frequency Questionnaire; MNA‐SF, Mini Nutritional Assessment‐Short Form; MUST, Malnutrition Universal Screening Tool; NRS‐2002, Nutritional Risk Screening 2002; PG‐SGA, Patient‐Generated Subjective Global Assessment; PNMS, Patient Nutrition Metabolic Score; SNAQ, Short Nutritional Assessment Questionnaire.

### 4.4 Requirements for Nurses in NNCs

Nurses in charge of consultations were required to be at the level of head nurse or above, accounting for 62.2%. Clinical specialty work experience of 6 years or more was required for 29.7% of them. A bachelor's degree or higher was required for 67.6% of them. Most hospitals required specialised nurses and further education for NNC nurses, as detailed in Table 5.

**TABLE 5:** Qualification requirements for nurses in NNCs.

Category	Frequency (*n*)	Percentage (%)
Professional Title		
Staff Nurse	7	18.9
Nurse Practitioner	7	18.9
Senior Nurse Supervisor	23	62.2
Clinical Experience (Years)		
1–3 years	4	10.8
4–5 years	13	35.1
6–10 years	8	21.6
> 10 years	3	8.1
No specific requirement	9	24.3
Education Level		
Technical Secondary School	1	2.7
College Diploma	6	16.2
Bachelor's Degree or above	25	67.6
No specific requirement	5	13.5
External Training Requirements		
National training programs	8	21.6
Training in tertiary hospitals	18	48.6
Training in secondary hospitals	2	5.4
No specific requirement	9	24.3
Nutrition Specialist Nurse Requirements		
Provincial‐level certification	17	45.9
Municipal‐level certification	7	18.9
No specific requirement	13	35.1

### 4.5 Current Status of Quality Monitoring Indicators in NNCs Based on Donabedian's Theory

Table 6 summarises the pool of quality monitoring indicators reportedly in use across the 37 NNCs, along with the frequency (*n*, %) of hospitals that reported using each indicator.

**TABLE 6:** Frequency of utilisation of quality monitoring indicators for NNCs.

Category	Indicator	Frequency	Percentage (%)
Structural Aspect			
Human Resources	Multidisciplinary team collaboration	19	51.35
	Proportion of specialised nutrition nurses	19	51.35
	Educational background	17	45.95
	Professional titles	18	48.65
	Years of work experience	22	59.46
	Research & innovation outputs (papers/conference presentations/patents/new technologies)	12	32.43
	Nursing competency (clinical decision‐making, nutritional risk assessment & intervention)	13	35.14
	Post status (full‐time/part‐time outpatient service)	13	35.14
Personnel Training	Standardised training certification rate	17	45.95
	Implementation rate of specialised nutrition training programs	17	45.95
Environment & Facilities	Clinic location arrangement (visibility vs. quietness)	8	21.62
	Clinic environment and layout	11	29.73
	Equipment functionality & availability	8	21.62
	Patient safety & privacy protection measures	7	18.92
Responsibility System	Nutritional Nursing Technical Operation Standard System	26	70.30
	Consultation System	20	54.10
	Follow‐up System	19	51.40
	Patient File Management System	4	10.80
	Nutritional Technical Operation Standard System	5	13.50
	Adverse Event Reporting System	24	64.90
	Nutritional Assessment and Management System	27	73.00
	Outpatient Appointment System	15	40.50
	New Technology/Project Introduction Access System	12	32.40
	Emergency Response Plan	10	27.00
	Nutritional Teaching System	2	5.40
	Quality Management System	12	32.40
	Qualification standards & job responsibilities for nutrition clinic staff	19	51.40
	Nutritional Nursing‐Related Hospital Infection Prevention and Control System	22	59.50
	Outpatient Clinic Management System	22	59.50
	Health Education System	12	32.40
	Continuing Education System	12	32.40
	Performance Evaluation System	10	27.00
	Satisfaction Survey System	12	32.40
	Internet+ Outpatient Service System	8	21.60
	Specialised Nurse Outpatient Service Filing System	7	18.90
	Nutritional Nursing Teaching System	11	29.70
Process Aspect			
Nutritional Assessment and Care	Comprehensiveness of nutritional assessment	26	70.27
	Accuracy & timeliness of nutritional assessment	20	54.05
	Individualised nutritional care plan formulation rate	23	62.16
	Pass rate of nutritional care technical assessments	19	51.35
	Popularisation rate of nutritional health education	22	59.46
	Compliance rate with standardised nutritional care operations	21	56.76
	Training Patients in Nutritional Knowledge, Behavioural Change and Self‐Management Skills	17	45.95
	Regularity & scientific validity of monitoring/adjustment protocols	14	37.84
	Policy implementation rate	15	40.54
	Scope compliance of implemented nutritional care techniques	14	37.84
	Interdisciplinary collaboration with healthcare professionals	14	37.84
Patient Record and Follow‐up Management	Completeness rate of outpatient medical records	2	5.41
	Patient file establishment	7	18.92
	Effectiveness of patient record management systems (EMR completeness)	11	29.73
	Frequency & quality of follow‐up evaluations	14	37.84
	Patient follow‐up rate	14	37.84
Outcome Aspect			
Patient Outcomes	Nutritional status improvement rate (weight/BMI/muscle mass/grip strength/6 m‐walk speed/serum albumin)	13	35.14
	Quality of life improvement rate	10	27.03
	Patient compliance rate	14	37.84
Incidence of Safety (Adverse) Events	Incidence Rate of Hospital‐Acquired Infections Related to Nutritional Nursing	17	45.95
	Complication rate (hospitalisation/relapse due to improper intervention)	19	51.35
	Adverse event reporting rate in nutritional interventions	21	56.76
Satisfaction	Patient satisfaction rate	16	43.24
	Nurse practitioner satisfaction rate	13	35.14
	Collaborating physician satisfaction rate	9	24.32
	Nutrition clinic complaint incidence	19	51.35
Health Economic Benefits	Quality control sensitive indicator monitoring	18	48.65
	Diagnostic service coverage	6	16.22
	Total cost of nutritional care services	7	18.92
	Economic benefits	10	27.03

### 4.6 Other Suggestions

Two hospitals provided suggestions for the development of nutrition clinics. One hospital suggested strengthening publicity and departmental cooperation, while the other indicated that NNCs should first be able to generate revenue and survive and could form upstream and downstream relationships with other departments.

## 5 Discussion

### 5.1 The Importance of NNCs

NNCs provide essential outpatient services, including assessment, consultation and education, led by specialised nutrition nurses (Cao et al. 2023). Evidence shows they address post‐discharge nutrition concerns, reduce complications, improve patient satisfaction and yield significant patient benefits (Huang et al. 2016).

### 5.2 Analysis of the Current Status of NNCs

#### 5.2.1 Development Status, Foundational Capacity and Operational Models

##### 5.2.1.1 Development Status and Growth Trajectory

Survey data from 113 hospitals nationwide indicate a low NNC establishment rate of 32.7%, signifying substantial room for expansion. The operational duration of existing clinics varies widely (from < 1 to 12 years), reflecting the field in different stages of maturity. Notably, 75.7% of NNCs were established within the last 5 years, pointing to rapid recent growth. This trend is likely driven by increased public health awareness, the rising burden of chronic diseases and a healthcare shift towards health management, highlighting both the potential and challenges for NNC development in China, particularly regarding expansion beyond tertiary hospitals (Wang and Zhao 2023).

##### 5.2.1.2 Foundational Capacity: Constraints and Professionalisation

Data from the 37 established NNCs reveal constraints in physical resources and operational scale. Service frequency is limited, with 78.4% operating 1–3 days weekly and patient throughput is modest (≤ 15 daily consultations for 75.6% of NNCs). This suggests most clinics function on a small scale, which may indicate either focused care models or underutilisation. Enhancing service frequency and capacity is a recognised development need (Cao et al. 2023). Concurrently, a trend towards professionalisation is evident. Most NNCs (70.3%) are managed by nursing departments and 51.4% employ full‐time nurses. Personnel standards are generally high, with most nurses holding senior titles (62.2%) and bachelor's degrees or higher (67.6%) and a significant proportion being certified specialist nurses (64.8%). However, the lack of unified standards is suggested by the 24.3% of NNCs reporting no clear qualification requirements.

##### 5.2.1.3 Service Models, Scope and Patient Access

Service provision is diversifying in mode and scope. While traditional in‐person consultations remain common (100%), 40.5% of NNCs have integrated online services. However, fully integrated models (in‐person, online and home services) are limited (10.8%), representing a key area for development to enable continuous, personalised care (Mi et al. 2018). The scope of services is broad, with all NNCs providing core activities (screening, assessment, education). Technical procedures (e.g., EN/PN management) are prevalent (70.3%), as are personalised nutrition advice (73.0%) and chronic disease management (48.7%). In contrast, services requiring extended coordination, like out‐of‐hospital management (29.7%), are less common. Patient access primarily involves direct registration (62.2%) and doctor referrals (56.8%), with word‐of‐mouth (40.5%) and community outreach (35.1%) also contributing significantly, underscoring the importance of both professional networks and public promotion.

#### 5.2.2 Professional Practice Content, Tools and Service Quality

##### 5.2.2.1 Equipment and Nutritional Assessment Tools

NNCs are well‐equipped with fundamental devices: weight scales (91.9%), height scales (89.2%) and basal metabolic rate metres (81.1%) are widely available. The adoption of more advanced tools like Bioelectrical Impedance Analysers (BIA) for body composition analysis is moderate (43.2%). A diverse array of assessment tools is utilised. General screening relies heavily on tools like the NRS‐2002 (59.5%) and basic anthropometric measurements (67.6%). For in‐depth evaluation, specialised tools such as PG‐SGA (Patient‐Generated Subjective Global Assessment, 37.8%) and MNA‐SF (Mini‐Nutritional Assessment Short‐Form, 40.5%) are employed for specific populations, such as cancer patients and the elderly. The existence, though with lower utilisation, of tools like the Paediatric Nutritional Risk Screening (PNMS, 18.9%) indicates capacity for tailored assessments.

##### 5.2.2.2 Health Education and Intervention Methods

Health education methods are diverse. Traditional methods dominate, with oral education (94.6%) and brochures (91.9%) being the most common. The integration of digital and interactive methods is growing, including video education (70.3%), which is effective for demonstrating practical content (Cao et al. 2023), telephone/online consultations (51.4%) and the use of social media/applications (43.2%). This variety allows adaptation to different patient needs and preferences.

##### 5.2.2.3 Service Portfolio: Scope, Depth and Identified Gaps

The service profile is comprehensive and professional. Core clinical activities—including nutrition screening, assessment and education—are universally offered (100%). Technical nursing skills, such as enteral/parenteral nutrition (EN/PN) pathway management, are prevalent (70.3%). Services like providing personalised medical diets/advice (73.0%) and chronic disease management (48.7%) are commonly provided. However, services requiring extended care coordination beyond the clinic setting, notably out‐of‐hospital nutrition management (29.7%), are significantly less common. This contrast reveals a stronger institutional emphasis on immediate, in‐clinic interventions compared to long‐term, coordinated care management, identifying continuity of care as a key area for development, a perspective supported by existing literature (Ding et al. 2017).

#### 5.2.3 Critical Developmental Barriers and Systemic Challenges

The survey identifies a set of interconnected barriers that threaten the sustainability and growth of NNCs, spanning policy, finance, professional authority and operational domains.

##### 5.2.3.1 Policy and Financial Barriers

The most frequently reported challenges pertain to financial sustainability. The absence of specific billing items for nutrition nursing techniques (67.6%) and the lack of inclusion of these services in medical insurance reimbursement (59.5%) are primary concerns. This dual challenge discourages institutional investment in NNCs and increases the financial burden on patients, potentially limiting service utilisation and long‐term viability.

##### 5.2.3.2 Professional Authority and Recognition Bottlenecks

A significant gap exists between policy intent and clinical practice regarding professional autonomy. While national policies support the expansion of nurses' roles (National Health Commission of the People's Republic of China 2022b; The First Hospital of Shanxi Medical University et al. 2018), the survey reveals limited implementation of nurse prescription rights. Collaborative prescribing under a doctor's guidance is most common (54.0%). Only 29.7% of NNC nurses have independent non‐pharmacological prescription rights and a mere 5.4% have independent pharmacological rights. Furthermore, 10.8% of NNC nurses have no prescription rights at all, a situation attributed to disparities in training, institutional support and local policies. This lack of authority remains a major practical difficulty for nurses in specialty clinics (Ding et al. 2017), despite being recognised as a key strategy to meet service demands (Cheng and Xia 2017). Compounding this is the low public and professional awareness of NNCs (46.0%), which hinders patient access and the full integration of nutrition nursing into the healthcare system.

##### 5.2.3.3 Operational and Collaborative Obstacles

Operational inefficiencies and systemic fragmentation further impede development. NNCs face difficulties in securing a stable patient base (limited sources reported by 37.8% of clinics) and fostering effective multidisciplinary collaboration (ineffective collaboration reported by 27.0%). Challenges in providing personalised services (18.9%), addressing cultural/dietary differences (24.3%), and—most critically—establishing effective follow‐up and continuity of care mechanisms (difficulties reported by 40.5%) underscore the struggle to manage long‐term patient needs beyond the initial clinic consultation. Resolving these multifaceted challenges requires concerted efforts in policy reform, education, technological investment and cross‐professional collaboration.

#### 5.2.4 Current Status of Quality Monitoring Indicators: Heterogeneity, Focus Areas and Implications for Future Framework Development

This study provides the first systematic mapping of quality monitoring indicators used by NNCs in China, revealing a heterogeneous landscape with distinct patterns of focus that inform future framework development.

##### 5.2.4.1 Heterogeneity

A key finding is the substantial heterogeneity in indicator adoption across the 37 NNCs. The utilisation frequency of the 66 identified indicators (Table 6) varied widely, reflecting a lack of national standardisation. While partly due to local adaptations, this variation presents a barrier to benchmarking and dissemination of best practices (Stanley et al. 2020).

##### 5.2.4.2 Patterns of Focus Across SPO Dimensions

Within this heterogeneous landscape, distinct patterns of focus emerge when examining the structure, process and outcome dimensions separately. Structure: Monitoring concentrates on core human resources and technical standards, with less focus on the clinic environment, safety infrastructure and research innovation. Process: Focus is stronger on initial care phases (assessment, planning) than on continuity. Low monitoring of record completeness, follow‐up protocols and interdisciplinary collaboration indicates an emphasis on initial intervention over continuous management. Outcome: Monitoring is oriented towards a risk‐management model (tracking adverse events), with under‐monitoring of positive value indicators like patient‐reported quality of life and health economic benefits.

##### 5.2.4.3 Implications for Developing a Standardised Quality Evaluation Framework

The heterogeneity underscores the need for a unified framework. The observed patterns provide direct guidance:

- A future framework must define clear measurement methods and benchmarks.
- It should integrate currently under‐monitored indicators, particularly those related to care continuity, patient‐reported outcomes and health economics (Chakraborty 2018; Guha et al. 2024).
- Its design must balance standardisation with local adaptability.

In conclusion, this study maps the pre‐standardisation terrain. The identified indicators serve as an evidence‐based repository for the next phase: developing and validating a standardised tool to enable systematic quality improvement.

### 5.3 Research Limitations

This study has several limitations. The sample of 113 hospitals across 41 cities may not fully represent all regions of China. Furthermore, the sample exhibited a pronounced concentration in tertiary hospitals (76.1%) and eastern regions (east‐to‐other regions ratio: 1.3:1), a pattern reflecting yet also potentially reinforcing the current skewed distribution of medical resources. This limits the generalisability of findings, particularly regarding developmental constraints and the state of nutritional care services in primary healthcare institutions. The reliance on self‐reported survey data also carries a risk of reporting bias. Finally, the study did not deeply explore specific contextual factors, such as individualised patient needs or cultural differences, that may influence service quality and satisfaction. Future research should employ a multi‐tiered sampling framework that intentionally includes primary healthcare institutions and community settings to obtain a more holistic assessment of the NNC ecosystem in China.

## Author Contributions

All authors contributed to the study conception and design. Preparation of materials, data collection and analysis were performed by Xiuya Ren, Xuan Ni and Xing Zeng. The initial draft of the manuscript was written by Yajing Gu, Hui Liu and Xing Zeng. All authors provided valuable input and feedback on previous versions of the manuscript. All authors read and approved the final manuscript. Funding and resource acquisition was coordinated by Xing Zeng and Xuan Ni. The quality of the research was supervised by Zhili Shen, Hui Liu and Ai Li. Project administration was managed by Changdi Li. All participants provided signed informed consent for the publication of their data.

## Funding

This study was supported by grants from the Nanjing Health Science and Technology Development Project (Youth Talent Science and Technology Project, QNX25108), the Chinese Hospital Reform and Development Institute at Nanjing University (NDYG2023057) and the Project of “Nursing Science” Funded by the 4th Priority Discipline Development Program of Jiangsu Higher Education Institutions (Jiangsu Education Department (2023) No. 11).

## Ethics Statement

This study is a cross‐sectional survey that solely analysed anonymised institutional‐level data, including information on resource allocation, service processes and quality indicators. According to Article 32, Paragraph 2 of China's ‘Ethical Review Measures for Life Sciences and Medical Research Involving Human Subjects’ (2023), research utilising anonymised information that does not cause harm to individuals and does not involve sensitive personal information may be exempt from ethical review.

## Consent

This multicentre cross‐sectional survey collected anonymised administrative data and service implementation status information from healthcare institutions. No personally identifiable patient information, medical records, or biological specimens were involved. In accordance with the Declaration of Helsinki and China's Ethical Review Measures for Biomedical Research Involving Humans, written informed consent was waived because only anonymised institutional data were used.

## Conflicts of Interest

The authors declare no conflicts of interest.

## Data Availability

The data that support the findings of this study are available from the corresponding author upon reasonable request.
